# Supplementary material for: Modeling glioblastoma heterogeneity as a dynamic network of cell states
Source: Mol Syst Biol. 2021 Sep 16;17(9):e10105. doi: 10.15252/msb.202010105 (PMC8444284; doi:10.15252/msb.202010105)
Supplement: Supplementary file 5 — Source Data for Figure 3 [file MSB-17-e10105-s001.zip › Figure3A_sourcedata/GSEA_3065/nosigdb_state4.GseaPreranked.1622718430994/SUVA_G2M_PMID_31327527.html]

Details for gene set SUVA\_G2M\_PMID\_31327527[GSEA]

|  || Dataset | state4 |
| Phenotype | NoPhenotypeAvailable |
| Upregulated in class | na\_neg |
| GeneSet | SUVA\_G2M\_PMID\_31327527 |
| Enrichment Score (ES) | -0.87703866 |
| Normalized Enrichment Score (NES) | -2.7216094 |
| Nominal p-value | 0.0 |
| FDR q-value | 0.0 |
| FWER p-Value | 0.0 |
Table: GSEA Results Summary

  

Fig 1: Enrichment plot: SUVA\_G2M\_PMID\_31327527      
 Profile of the Running ES Score & Positions of GeneSet Members on the Rank Ordered List

  

| PROBE | GENE SYMBOL | GENE\_TITLE | RANK IN GENE LIST | RANK METRIC SCORE | RUNNING ES | CORE ENRICHMENT || 1 | PSRC1 |  |  | 2990 | 0.025 | -0.4787 | No |
| 2 | KIF20A |  |  | 5260 | -0.107 | -0.8351 | No |
| 3 | RANGAP1 |  |  | 5463 | -0.127 | -0.8577 | No |
| 4 | HMGB3 |  |  | 5584 | -0.142 | -0.8660 | Yes |
| 5 | KNSTRN |  |  | 5610 | -0.146 | -0.8587 | Yes |
| 6 | CDCA8 |  |  | 5642 | -0.151 | -0.8520 | Yes |
| 7 | KIFC1 |  |  | 5645 | -0.152 | -0.8405 | Yes |
| 8 | TTK |  |  | 5684 | -0.157 | -0.8344 | Yes |
| 9 | GPSM2 |  |  | 5739 | -0.169 | -0.8299 | Yes |
| 10 | REEP4 |  |  | 5749 | -0.172 | -0.8180 | Yes |
| 11 | NCAPD2 |  |  | 5778 | -0.179 | -0.8086 | Yes |
| 12 | PLK1 |  |  | 5836 | -0.192 | -0.8028 | Yes |
| 13 | CKAP2 |  |  | 5879 | -0.201 | -0.7940 | Yes |
| 14 | SPAG5 |  |  | 5899 | -0.207 | -0.7810 | Yes |
| 15 | BUB1B |  |  | 5901 | -0.207 | -0.7650 | Yes |
| 16 | RACGAP1 |  |  | 5953 | -0.223 | -0.7559 | Yes |
| 17 | TROAP |  |  | 5983 | -0.232 | -0.7425 | Yes |
| 18 | MZT1 |  |  | 5989 | -0.235 | -0.7251 | Yes |
| 19 | KIF2C |  |  | 5996 | -0.237 | -0.7076 | Yes |
| 20 | AURKA |  |  | 6026 | -0.248 | -0.6930 | Yes |
| 21 | MXD3 |  |  | 6042 | -0.265 | -0.6748 | Yes |
| 22 | BUB1 |  |  | 6045 | -0.266 | -0.6544 | Yes |
| 23 | TUBA1C |  |  | 6052 | -0.270 | -0.6344 | Yes |
| 24 | ARHGAP11A |  |  | 6067 | -0.277 | -0.6151 | Yes |
| 25 | FOXM1 |  |  | 6070 | -0.279 | -0.5938 | Yes |
| 26 | CCNA2 |  |  | 6100 | -0.297 | -0.5754 | Yes |
| 27 | CKAP2L |  |  | 6108 | -0.303 | -0.5529 | Yes |
| 28 | KIF11 |  |  | 6109 | -0.304 | -0.5293 | Yes |
| 29 | TACC3 |  |  | 6138 | -0.328 | -0.5082 | Yes |
| 30 | CCNB2 |  |  | 6140 | -0.329 | -0.4828 | Yes |
| 31 | ARL6IP1 |  |  | 6145 | -0.336 | -0.4573 | Yes |
| 32 | CDC20 |  |  | 6154 | -0.344 | -0.4319 | Yes |
| 33 | KIF4A |  |  | 6164 | -0.355 | -0.4058 | Yes |
| 34 | CDCA3 |  |  | 6165 | -0.355 | -0.3782 | Yes |
| 35 | ANP32E |  |  | 6168 | -0.357 | -0.3508 | Yes |
| 36 | ECT2 |  |  | 6178 | -0.382 | -0.3225 | Yes |
| 37 | TUBB4B |  |  | 6196 | -0.431 | -0.2917 | Yes |
| 38 | SMC4 |  |  | 6219 | -0.490 | -0.2572 | Yes |
| 39 | TMPO |  |  | 6233 | -0.522 | -0.2187 | Yes |
| 40 | KPNA2 |  |  | 6236 | -0.543 | -0.1768 | Yes |
| 41 | CCNB1 |  |  | 6242 | -0.606 | -0.1305 | Yes |
| 42 | H2AFZ |  |  | 6250 | -0.651 | -0.0811 | Yes |
| 43 | CENPF |  |  | 6263 | -1.068 | -0.0000 | Yes |
Table: GSEA details [plain text format]

  

Fig 2: SUVA\_G2M\_PMID\_31327527: Random ES distribution      
 Gene set null distribution of ES for **SUVA\_G2M\_PMID\_31327527**
